# Lack of Genotype and Phenotype Correlation in a Rice T-DNA Tagged Line Is Likely Caused by Introgression in the Seed Source

**DOI:** 10.1371/journal.pone.0155768

**Published:** 2016-05-17

**Authors:** Fu-Jin Wei, Yuan-Ching Tsai, Yu-Ming Hsu, Yu-An Chen, Ching-Ting Huang, Hshin-Ping Wu, Lin-Tzu Huang, Ming-Hsin Lai, Lin-Yun Kuang, Shuen-Fang Lo, Su-May Yu, Yann-Rong Lin, Yue-Ie Caroline Hsing

**Affiliations:** 1 Institute of Plant and Microbial Biology, Academia Sinica, Taipei, Taiwan; 2 Department of Agronomy, National Taiwan University, Taipei, Taiwan; 3 Crop Science Division, Taiwan Agriculture Research Institute, Taichung, Taiwan; 4 Transgenic Plant Core Facility, Academia Sinica, Taipei, Taiwan; 5 Institute of Molecular Biology, Academia Sinica, Taipei, Taiwan; Oregon State University, UNITED STATES

## Abstract

Rice (*Oryza sativa*) is one of the most important crops in the world. Several rice insertional mutant libraries are publicly available for systematic analysis of gene functions. However, the tagging efficiency of these mutant resources–the relationship between genotype and phenotype–is very low. We used whole-genome sequencing to analyze a T-DNA–tagged transformant from the Taiwan Rice Insertional Mutants (TRIM) resource. The phenomics records for M0028590, one of the TRIM lines, revealed three phenotypes–wild type, large grains, and tillering dwarf–in the 12 T_1_ plants. Using the sequencing data for 7 plants from three generations of this specific line, we demonstrate that introgression from an *indica* rice variety might occur in one generation before the seed was used for callus generation and transformation of this line. In addition, the large-grain trait came from the *GS3* gene of the introgressed region and the tillering dwarf phenotype came from a single nucleotide change in the *D17* gene that occurred during the callus induction to regeneration of the transformant. As well, another regenerant showed completely heterozygous single-nucleotide polymorphisms across the whole genome. In addition to the known sequence changes such as T-DNA integration, single nucleotide polymorphism, insertion, deletion, chromosome rearrangement and doubling, spontaneous outcrossing occurred in the rice field may also explain some mutated traits in a tagged mutant population. Thus, the co-segregation of an integration event and the phenotype should be checked when using these mutant populations.

## Introduction

Rice (*Oryza sativa*) is one of the most important crops in the world. With the increasing world population, we need to feed people in a more sustainable and environmentally friendly way. With the complete genome sequencing of rice [1], the challenge of the post-genomic era is to systematically analyze the functions of all rice genes. One way to define the function of a novel gene is to knock out and/or activate its expression. Insertional mutagenesis by using T-DNA [2–6] or transposable elements such as *Tos17* [7], *Ac/Ds* [8–11] or *Spm* [12] provides an efficient way to isolate the target gene responsible for a specific phenotype. Many research groups have established rice insertional mutant resources and provided flanking sequence tag (FST) information for these lines [13–15].

However, these rice insertional mutant libraries have low tagging efficiency; that is, the relationship between genotype (disrupted or activated gene) and phenotype is very low. For forward genetic screens, the estimated tagging efficiency is 5% to 10% in *Tos17*- and T-DNA–tagged populations [13, 16]; the efficiency is ~35% in *Arabidopsis* T-DNA–tagged insertional populations [17, 18], much higher than in rice mutants. In both of these species, many observed phenotypes are not caused by the integration of T-DNA or the transposon, so other sequence changes are responsible.

Using T-DNA as a vector with a local *japonica* rice variety, Tainung 67 (TNG67), we prepared a huge rice insertional mutant resource in Taiwan. The collection was designated Taiwan Rice Insertion Mutants (TRIM) and is accessed at http://trim.sinica.edu.tw. The vector we used provided three functions: knock-out, activation, and promoter trapping [5]. We cooperated with rice breeders who grew and evaluated plants and thus obtained detailed phenomics information, including 11 categories or 69 subcategories, for 12 T_1_ plants per line grown in a genetically modified (GM) field; the data are available in the TRIM database [19]. As of early spring 2016, about 120,000 lines have been generated from the tissue culture lab; about 73,000 lines have phenomics data and 65,000 have FST data. The seeds are available upon request at the T-DNA Tagged Rice Service Center (http://tdna.bts.asia.edu.tw/) [5].

The data have been used for several forward genetics analyses, finding that *OsGA2ox* plays important roles in rice root and shoot architecture [20], that abolishment of a SUMO E2 ligase causes anther dehiscence [21], that abolishment of a bHLH transcription factor causes malfunction of tapetum and thus male sterile [22], and that activation of a RING Zinc finger protein enhances stomata opening [23]. *Tos17* in TNG67 is quite stable and does not transpose [5], so the tagging efficiency of the TRIM population is greater than most of the other insertional mutant resources [24].

With the phenomics data, we started working on the genes that may increase rice yield. One of the important traits is rice big grain (RBG). There are several putative RGB traits in the TRIM resource. We studied RBG1 to RBG3 and demonstrated the genes responsible for the BG by using an overexpression strategy [24]. However, M0028590, one of the TRIM lines featuring BG did not co-segregate with the T-DNA integration.

Sequence changes in the transformants may be caused by callus growth, the *Agrobacterium* incubation medium, virulence genes, transformation, and selection conditions. We previously used high-throughput sequencing of DNA from rice lines derived from TNG67 to analyze non-transformed and transgenic rice plants for mutations caused by these conditions [25]. For comparison, we also analyzed sequence changes for two additional rice varieties and four TRIM lines. We identified single nucleotide polymorphisms (SNPs), small indels, large deletions, chromosome doubling, and chromosome translocations in these accessions. The SNPs and indel frequencies of regenerants and transformants, normalized to the event rate at R_0_ or T_0_ generation, were about 200 events per plant. Using three to four duplicates for each treatment, we found no significant difference between any of these treatments. The frequency of TRIM lines was a bit higher, from 550 to 1300 per T_0_ plant [25].

In the present study, we chose M0028590, one TRIM line with three distinct phenotypes–wild type, large grains, and dwarf and high tillering number–including seven T_1_, T_2_, and T_3_ plants, and performed whole-genome sequencing to find sequence changes responsible for the phenotypes. T-DNA integration did not contribute to the phenotypes. In addition, the SNP frequency was much higher with this TRIM line than other TRIM lines. We found that introgression from an *indica* rice variety must have occurred. In addition, introgression and SNPs may have explained the two mutated traits.

## Materials and Methods

### Plant material and regeneration

A TRIM line M0028590 was chosen for study because the phenomics data for its T_2_ plants showed three different phenotype traits. Although more than three-quarters of the TRIM mutants show knocked-out and activation functions, the Tag4 construct [5] was used as the vector for this specific line, and thus no 35S enhancer was present in the T-DNA. Therefore, this TRIM line showed only a knock-out function. The T_1_ and T_2_ seeds for the transformant M0028590 were obtained from the TRIM resource center [5, 19]. The T_3_ seeds were propagated from T_2_ plants by a single-seed–descent practice in a certified GM field.

To study the mechanism of the low tagging efficiency, we generated several regenerants and transformants for further whole-genome sequencing. One of the plants, Regenerant R (R_0_), was used for the current study. We followed the protocol of Sallaud, Meynard [26], with some modifications. The process was similar to that for transformation, except for no co-culture with *Agrobacterium*. In brief, the embryo of mature *japonica* variety TNG67 seeds was used to induce callus, which was then incubated at 25°C for 2 weeks under darkness. The embryogenic nodular callus was then transferred to liquid co-culture medium for 10 min without *Agrobacterium* cells. Callus pieces were then incubated on solid co-culture medium at 25°C under darkness for 3 days before being placed on selection medium with cefotaxime and incubated at 25°C under darkness for 5 weeks. Callus pieces were then placed on pre-regeneration medium for 1 week before being transferred onto regeneration medium for 3 weeks with 16-hr light per day. The shoots regenerating from callus were then dissected and sub-cultured on rooting medium. All media used were the same as for Sallaud, Meynard [26].

All plants were cultivated for 40 days in an Academia Sinica greenhouse under natural light. Healthy leaves without insect damage were harvested, frozen under liquid nitrogen and stored at -80°C.

### Genomic DNA extraction and sequencing

Genomic DNA was extracted from healthy leaves of a single-seed–descent plants by using the DNeasy Plant Mini Kit (Qiagen). After quality assessment, the genomic DNA was randomly fragmented and size-fractionated. DNA fragments with the desired lengths were gel-purified. For whole-genome resequencing, paired-end libraries with 450- to 500-bp inserts were constructed and sequenced with use of a GA2 or HiSeq2000 system (Illumina). Sequence data were deposited in the NCBI Sequence Read Archive (accession numbers are in Table 1).

**Table 1:** Summary of the sequencing data and plant materials.

	Read type^a^	Estimated average depth^b^	Genome coverage ratio	Accession no.	Characters of plants
**Tainung 67 (TNG67)**	101PE	25.8	97.5%	SRR1531575	An elite modern *japonica* variety.
**M0028590 T**_**1**_**a (T**_**1**_**a)**	101PE	14.2	97.2%	SRR2079330	One of the T_1_ plants from the M0028590
**M0028590 T**_**1**_**b (T**_**1**_**b)**	101PE	14.2	96.8%	SRR2079331	One of the T_1_ plants from the M0028590.
**M0028590 T**_**1**_**c (T**_**1**_**c)**	101PE	14.8	96.7%	SRR2079332	One of the T_1_ plants from the M0028590.
**M0028590 T**_**2**_**a (T**_**2**_**a)**	119PE	11.4	97.4%	SRR2079333	One of the T_2_ plants from the M0028590
**M0028590 T**_**2**_**b (T**_**2**_**b)**	119PE	12.2	96.0%	SRR2079335	One of the T_2_ plants from the M0028590
**M0028590 T**_**3**_**a (T**_**3**_**a)**	101PE	51.2	98.2%	SRR2079336	One of the T_3_ plants from the M0028590
**M0028590 T**_**3**_**b (T**_**3**_**b)**	101PE	65.1	97.8%	SRR2079338	One of the T_3_ plants from the M0028590
**Regenerant R**	126PE	18.8	98.0%	SRR2079339	A regenerant from Tainung 67.
**Taikeng 9 (TK9)**	75PE	6.4	94.0%	SRR2079340	An elite modern *japonica* variety.
**F**_**2**_ **wild-type pool**	101PE	13.4	97.9%	SRR2079341	Pools from 20 wild-type F2 population
**F**_**2**_ **big-grain pool**	101PE	16.4	98.8%	SRR2079342	Pools from 20 big-seed F2 population
**F**_**2**_ **big-grain plant 1**	101PE	14.9	97.1%	SRR2079343	A single big-seed F2 plant.
**F**_**2**_ **big-grain plant 2**	101PE	17.9	97.6%	SRR2079344	A single big-seed F2 plant.
**Taichung Sen 17 (TCS17)**	75PE	7.3	86.8%	SRR3098366	An elite modern *indica* variety.
**IR64**	126PE	30.5	91.5%	SRR3098100	An elite modern *indica* variety.
**Taichung 65 (TC65)**	75PE	6.9	93.1%	SRR1956774	A modern *japonica* variety.
**Taichung Sen (TCS10)**	126PE	17.1	89.6%	SRR3133264	An elite modern *indica* variety.

^a^ PE, paired end.

^b^ The estimated depth is the total number of bases divided by 400 M as a reference genome size.

### Mapping and variant calling

The paired reads were aligned to the reference rice Nipponbare genome sequence (IRGSP v1.0) by use of Burrows-Wheeler Aligner (BWA) [27]. SAMtools [28] and VCFtools [29] were used to manipulate and transform the sequence alignment/map format (SAM) and variant call format (VCF) [29] of the file. For mapping, “-q20” of the “aln” step was used to control base quality, with the files output as “Binary sequence Alignment/Map” (BAM). For variant calling, the default parameter value was used, and the variant records were output in VCF format. The VCF files for these lines were compared by “vcf-isec” to classify sample-specific or intersection variants. The VCF files were then imported into Integrative Genomics Viewer (IGV) [30, 31] to show alleles or genotypes. Further filtering was applied depending on the purpose, such as to identify the introgression regions. The VCF files for all analyzed plants were stored in the European Variation Archive (accession numbers shown in S1 Table).

### Identification of introgression regions

The high SNP-rate region of the TRIM line M0028590 from the wild-type TNG67 was initially estimated by an SNP plot of all 12 chromosomes. More precise borders were then narrowed down by manual visualization via IGV. The SNP locations in TNG67, the offspring of M0028590, another two TRIM lines without introgression, and one *indica* variety were investigated. The introgression border was defined as 10 kb away (5’ or 3’, depending on the orientation) from the last SNP position of any high-SNP-frequency regions on comparing the offspring of M0028590 and the control (TNG67 and the two other TRIM lines).

### Heterozygosity plot

The heterozygosity plot for Regenerant R was generated by use of the R package ggplot2 (http://ggplot2.org). The plots represented the SNP number per 100 kb of Regenerant R against the SNP positions of TNG67.

## Results

### M0028590 line showed three different phenotypes in T2 plants

**Fig 1:**
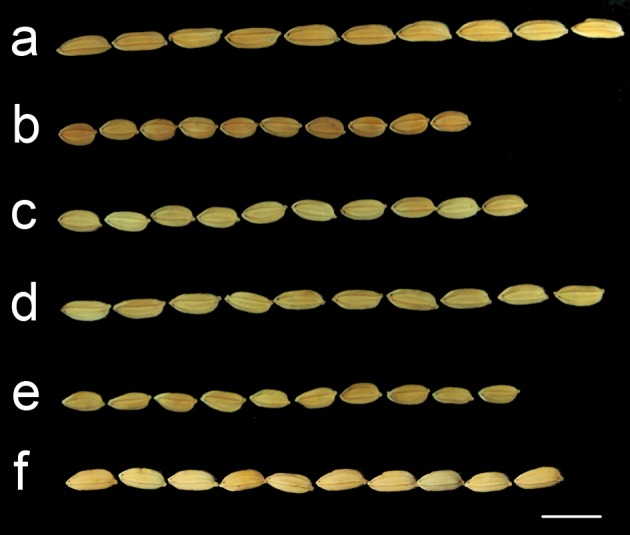
The phenotype of seeds used for the map-based cloning of the large-grain trait. Row a, a T_2_ large-grain plant–the female parental line; row b, TNG67 –the variety used to generate TRIM population; row c, TK9 –the male parental line; row d, large-grain F_2_ plant, row e, wild-type F_2_ plant; row f, T_1_c plant. n = 10 seeds each row. Scale bar: 1 cm.

We chose the TRIM line M0028590 for detailed analysis because the 12 T_1_ plants showed three different phenotypes and these phenotypes did not co-segregate with the T-DNA integration [19]. These plants were grown in the GM field during the second cropping season in 2006. We found three phenotypes according to the breeders’ record: (1) Group A. Wild type. Panicle per plant: 21, plant height: 112 cm, heading date: 73 days. (2) Group B. Large grain, long leaf. Panicle per plant: 18, plant height: 121 cm, heading date: 68 days. (3) Group C. Pale green and narrow leaf, erect and thin culm, semidwarf. Panicle per plant: 52, plant height: 75 cm, heading date: 71 days. Thus, group B had large grains and group C was a tillering dwarf mutant, with the characteristics of dwarfism and high number of tillers. However, the flowering time (i.e., heading date) for these groups was still the same. Fig 1 illustrates the grain size difference between group B plants (large grains, lane a) and the wild-type TNG67 (lane b).

### Rice genome re-sequencing

We re-sequenced the genome of two elite *japonica* rice varieties TNG67 and Taikeng 9 (TK9) as well as several offspring from the M0028590 plants: three T_1_ plants, including two with normal plant height (T_1_a and T_1_b) and one dwarf plant (T_1_c); two T_2_ plants, including one wild type (T_2_a) and one dwarf type (T_2_b); and two T_3_ plants (T_3_a and T_3_b, both dwarf type). We also sequenced a regenerant plant (Regenerant R) generated from TNG67. The sequencing type, data size, genome coverage ratio, accession numbers and general description of each accession are in Table 1. We grew these plants in the greenhouse and monitored the phenotypes until harvesting. The plant height, tiller numbers and grain characteristics of the varieties and lines used in the studies are all in Table 2. By observing several T_1_ and T_2_ plants as well as their offspring, we found that the dwarf tillering trait was heritable. The T_1_c plant was a tillering dwarf plant but contained large grains. Thus, large grain and tillering dwarf are two independent traits.

**Table 2:** Seed and plant traits for the plant materials used.

	Plant height*	Tiller number	Grain length*	Grain width*
**TNG67**	122.4±4.4	9.1±1.0	0.68±0.01	0.35±0.01
**TK9**	122.0±3.5	9.9±2.4	0.71±0.01	0.33±0.01
**Large grain**	127.8±4.1	11.4±2.4	0.85±0.01	0.32±0.01
**T_1_a**	118.2±3.3	10.3±2.5	0.74±0.01	0.33±0.01
**T_1_b**	117.5±4.1	11.1±1.9	0.72±0.01	0.34±0.01
**T_1_c**	67.8±6.8	46.6±6.4	0.83±0.01	0.32±0.01
**T_2_a**	119.2±3.9	11.3±2.5	0.74±0.01	0.34±0.01
**T_2_b**	65.8±5.9	45.5±5.8	0.72±0.01	0.33±0.01
**T_3_a**	68.1±6.6	47.6±5.4	0.73±0.01	0.33±0.01
**T_3_b**	66.8±7.1	46.8±5.7	0.72±0.01	0.33±0.01

*: unit in cm.

### High SNP rate in M0028590

The SNPs were estimated from the sequencing data without filtering, as indicated in Methods. The number of SNPs, including homozygous and heterozygous ones, with TNG67 was estimated at 392,059, 425,864 and 451,023 for the T_1_a, T_1_b and T_1_c plants, respectively. These numbers are much higher than for other transformants or TRIM lines, also generated from TNG67, that we studied previously [25]. To reveal the mechanism for such high SNP numbers, we compared *japonica* and *indica* rice varieties (S2 Table). The average SNP number for the *japonica* varieties usually grown in breeders’ fields in Taiwan was 188,520, and the average for randomly chosen 100 *japonica* accessions from the 3K project [32] was 1,125,990. However, the average for the *indica* rice varieties was 2,549,930 and 2,852,440, respectively. Thus, the SNP rates are very high for the M0028590 offspring.

### Part of the M0028590 contained an introgression region

**Fig 2:**
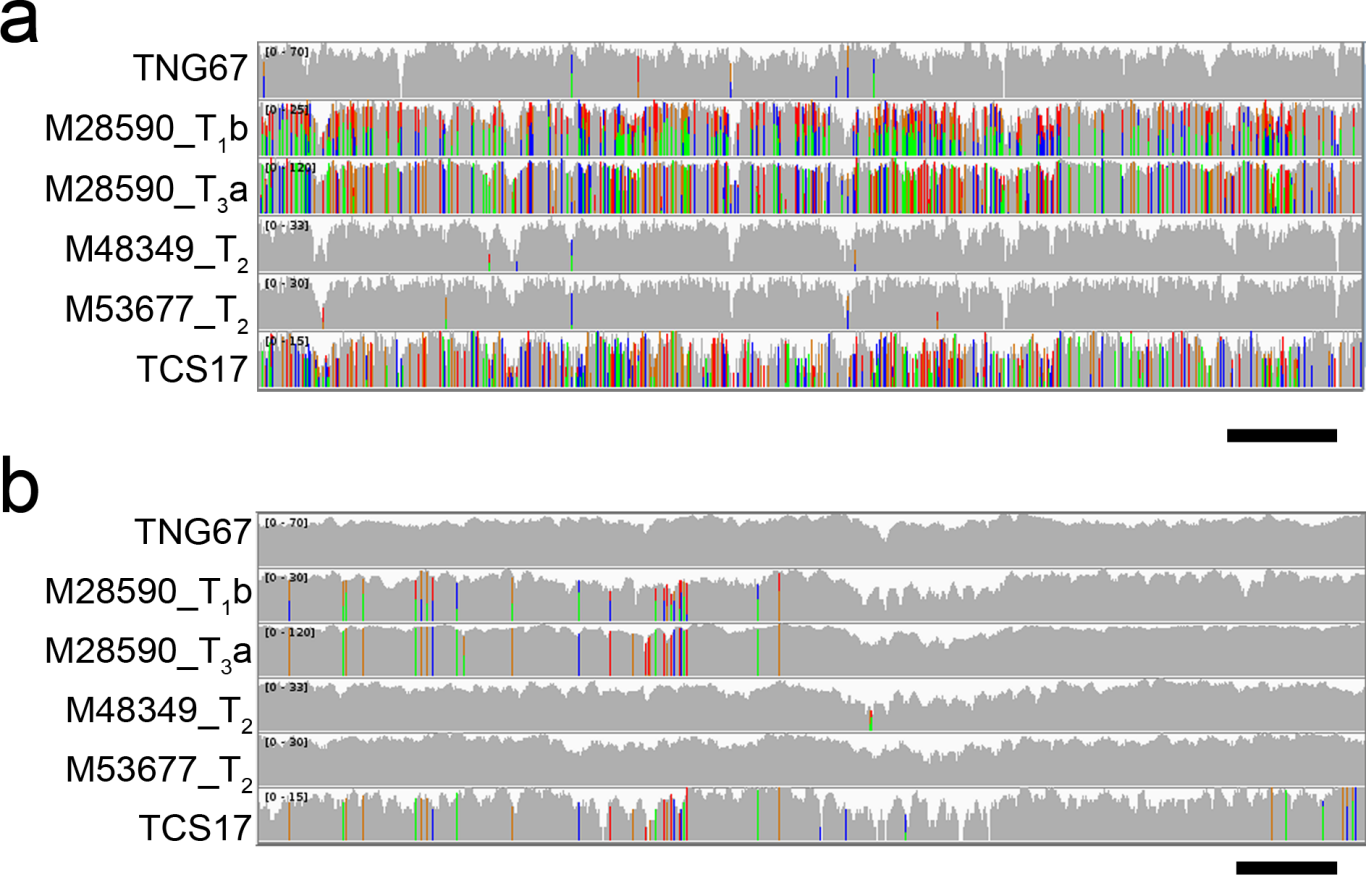
Introgression in M0028590 offspring. The Integrative Genomics Viewer (IGV) view of aligned reads. Panel a. A 100-kb region in chromosome 2 (35,200,001–35,300,000). The paired read sequencing was performed on an Illumina platform and the alignments were toward the Nipponbare IRGSP 1.0. The x-axis is the thickness of the read (shown in log scale), with the gray bar for matched reads (compared to Nipponbare); the mismatched reads for A are in green, G in orange, C in blue, and T in red. Rows a to f, Tainung 67 (TNG67), M0028590T_1_b, M0028590T_3_a, a T_2_ plant of M0048349, a T_2_ plant of M0053677, Taichungsen 17 (TCS17). Scale bar: 10 kb. Panel b. IGV view of aligned reads of a 10-kb region of chromosome 2 (35,420,601–35,431,600). Scale bar: 1 kb.

We then checked the distribution of these SNPs in the genome. If they scattered around the whole genome, it might be caused by an independent mutagenesis or artifact; if they located in certain regions, it might be caused by introgression from another variety. We tested the SNP distribution for seven offspring of M0028590, including three T_1_ plants, two T_2_ plants, and two T_3_ plants as well as other TRIM lines. Fig 2A illustrates a 100-kb region of chromosome 2. The gray bars indicate that the sequence is identical to the reference sequence. The mismatched bases are in green for A, orange for G, blue for C and red for T. The figure illustrates the SNP distribution for six accessions: TNG67, M0028590T_1_b, M0028590T_3_a, a T_2_ plant of M0048349, a T_2_ plant of M0053677, and TaichungSen17 (TCS17), an *indica* variety frequently grown in Taiwan. The SNP distribution for the other two TRIM lines was similar to that for TNG67, very few in the region. The high SNP number and location for the two M0028590 offspring were similar to that for TCS17. In addition, the SNPs were heterozygous in M0028590 T_1_b plants but homozygous in T_3_a plants. Fig 2B shows a zoom-in region locating 200 kb downstream from the region shown in Fig 2A. The two M0028590 offspring contain about 30 SNPs in the 5-kb region at the 5’ end and the sequence changes are similar to that for TCS17. Thus, the high frequency and similar localization of the SNP suggest that introgression from an *indica* rice might have occurred in this TRIM mutant line. In addition, sequencing the T_1_ and T_3_ generations revealed that segregation/recombination occurred.

**Fig 3:**
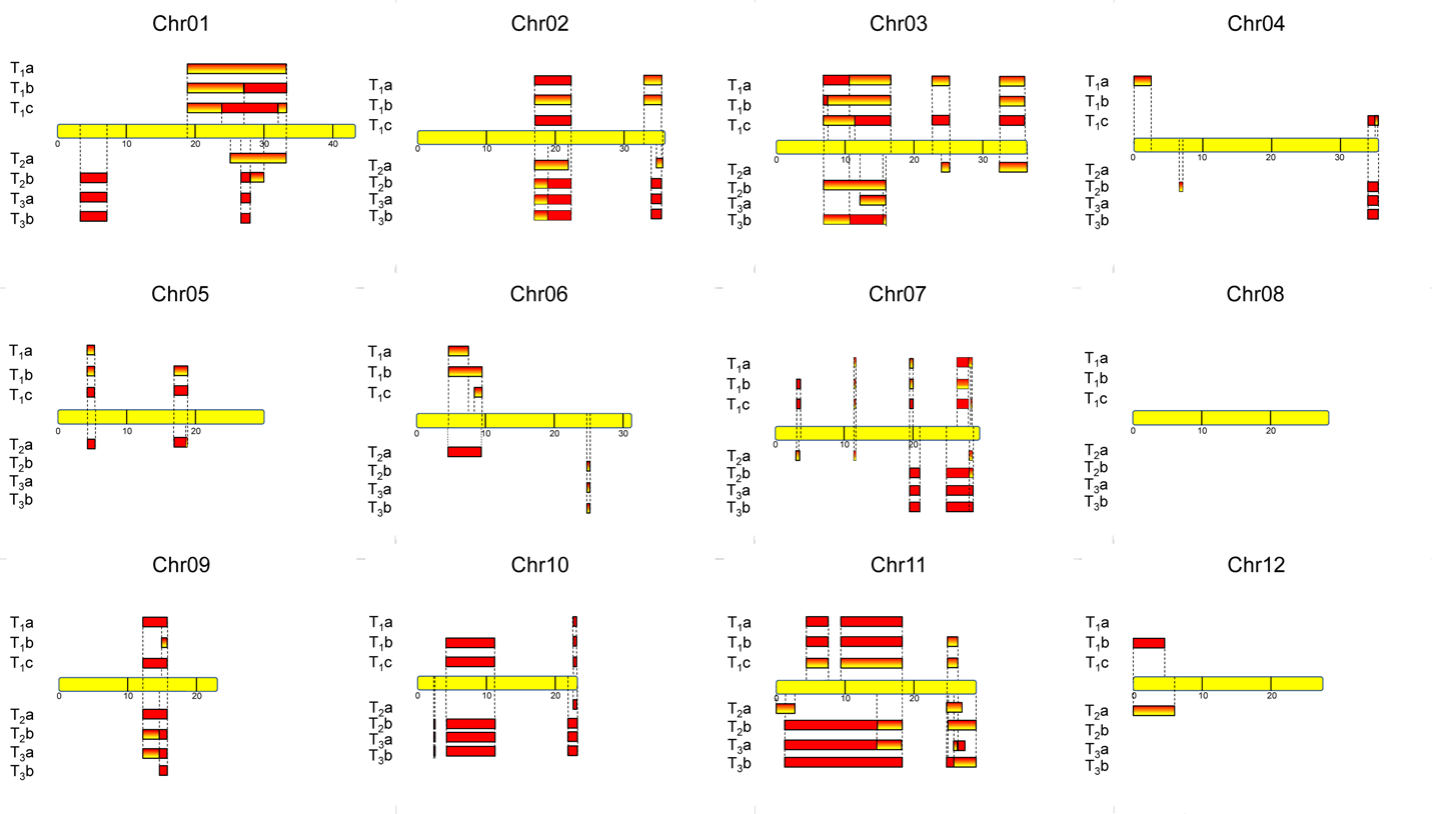
The introgression map of an *indica*-type genome into all chromosomes. Introgression into the 12 chromosomes of the TRIM line M0028590. Yellow bar is the TNG67 chromosome, and the numbers indicate the Mb in the chromosome. The introgression regions are revealed by SNPs of 7 plants, including 3 T_1_ plants, 2 T_2_ plants and 2 T_3_ plants. Gradient orange bars, regions with heterozygous SNPs; red bars, regions with homozygous SNPs.

We then checked the distribution of “high SNP frequency” regions of the 12 chromosomes for the seven M0028590 offspring and marked regions with homo- or heterozygous SNPs. Fig 3 shows the high SNP-frequency regions for all 12 chromosomes of the three generations. The heterozygous (orange gradient bar) or homozygous (red bar) SNP regions are present in certain regions, with the recombination in different generations easily seen. About three quarters of chromosome 11 contained such high SNP segments, with no such region in chromosome 8 for all T_1_, T_2_, and T_3_ plants.

**Fig 4:**
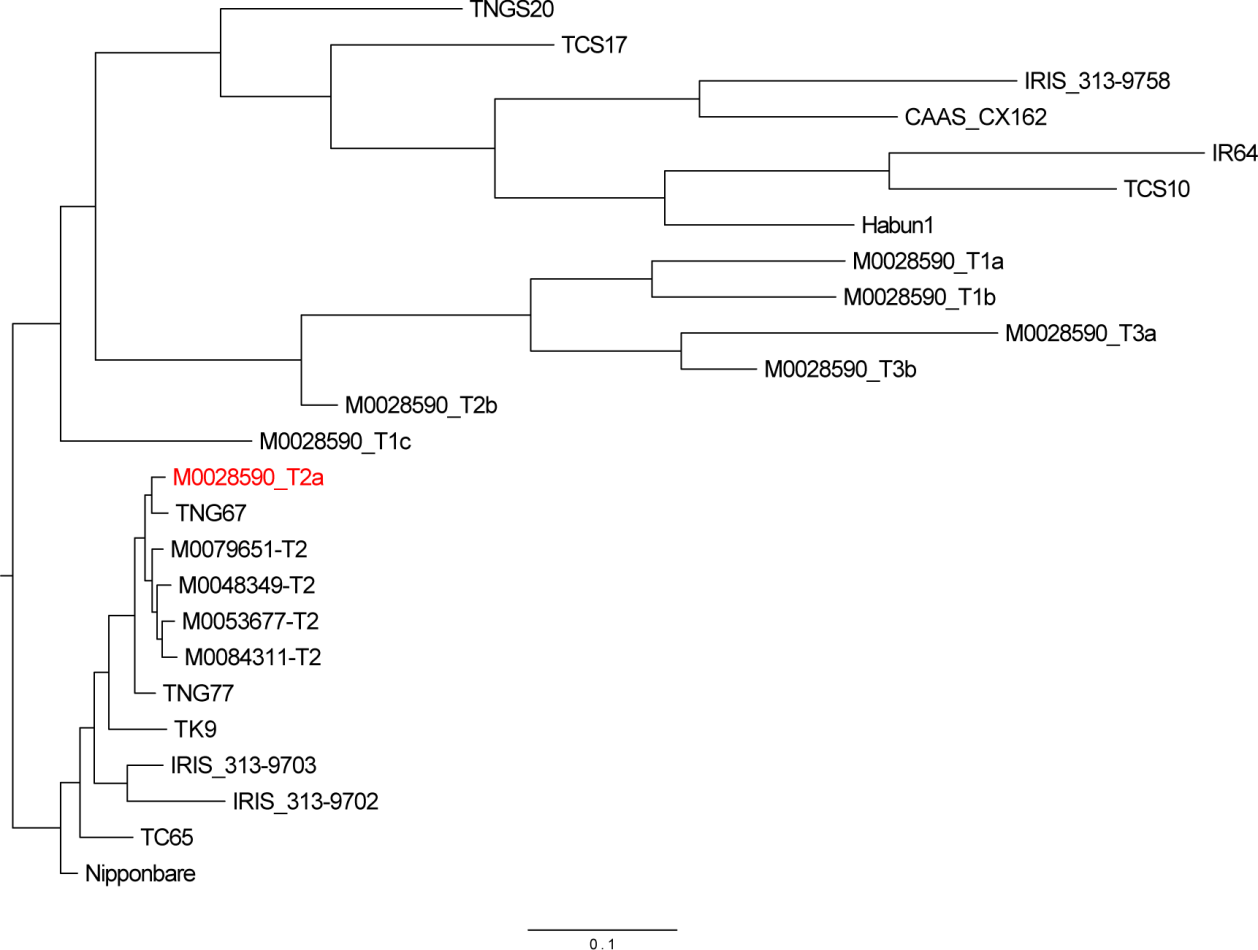
Phylogenic tree of M0028590 and other *japonica* and *indica* rice varieties. The tree was constructed with SNP data from 7 M0028590 offspring, 5 *indica*, 2 *Aus*, 7 *japonica* rice, and 4 TRIM mutant lines by using SNPhylo [34]. Only 18-Mb region where no introgression occurred in the T_2_a plant were used (S4 Table). The accession numbers and type of the 25 materials are in S3 Table.

To explore the reason for the high SNP rate in certain regions of the genome, we used dendrography. Since the seeds used to generate the TRIM lines were received from the rice breeders in Taiwan Agriculture Research Institute (TARI), we chose several *japonica*, *indica* and *Aus* varieties grown in Taiwan for comparison. The varieties include those we sequenced for current or previous studies [25, 33], as well as those from the 3K genome project [32]. The accession numbers of these lines are in Table 1 and S3 Table. Because T_2_a plants did not contain several “high-SNP” regions (Fig 3), we chose a 18-Mb region which the six other M0028590 offspring contained a high SNP rate not in T_2_a. Fig 4 shows that T_2_a (highlighted in red) is clustered with TNG67 and other *japonica* varieties, and the six other M0028590 offspring cluster with *indica* rice. We also checked their relationship by using whole-genome sequences (S1 Fig) and found that all seven M0028590 offspring clustered with *japonica* rice. Thus, the high SNP frequency region should be caused by introgression from an *indica* rice variety.

Because several of the M0028590 offspring showed homozygous introgression regions, we checked the consequences of the sequence changes. S5 Table summarizes the effects of SNPs on the homozygous introgression region of chromosome 1 in T_1_C plants. This 5-Mb region showed non-synonymous codons, frame shifts, splice sites, or early stop mutations, for altered amino acid sequences.

The introgression region for the TRIM line M0028590 from the wild-type TNG67 was initially estimated by an SNP plot of 5-kb sliding windows, with window size 2 Mb, across the 12 chromosomes (S2 Fig). The introgressed regions were then narrowed down by visualization, as indicated in Methods. Fig 2B shows an example of the border of an introgression event. This 10-kb region shows no SNP among TNG67 and the two TRIM lines M0048349 and M0053677 on chromosome 2. However, the left side of M0028590T_1_b, M0028590T_3_a and TCS17 showed a high number of SNPs. In addition, the location and type of these SNPs were similar among the three plants. However, on the right side, the SNPs were present only in TCS17. The introgression border was then defined as the point 10 kb away (3’ in this case) from the last SNP position of this introgression event. Using a similar strategy, we defined the borders of all introgression events in the seven plants (S6 Table). By using the information for the introgressed regions, we estimated the SNP numbers specifically occurring there (Table 3). For the seven offspring, the SNP numbers ranged from 193,099 to 399,390. Exactly in the same regions, the mean number of SNPs for the *japonica* varieties ranged from 13,314 to 41,565, but 271,877 to 528,347 SNPs for *indica* varieties. We sequenced about five *indica* varieties usually grown in breeders’ fields but did not find any with identical SNPs to the M0028590 offspring.

**Table 3:** Number of single nucleotide polymorphisms (SNPs) in the introgression regions.

	No. of SNPs in the introgressed regions*	Mean SNP no. in the introgressed regions‘	
		*Indica*	*Japonica*
**T**_**1**_**a**	347,426	458,296	26,731
**T**_**1**_**b**	393,367	523,397	31,548
**T**_**1**_**c**	399,390	509,259	31,225
**T**_**2**_**a**	193,099	271,877	13,314
**T**_**2**_**b**	276,047	422,045	31,592
**T**_**3**_**a**	321,813	528,347	41,565
**T**_**3**_**b**	319,522	504,645	39,842

The SNPs were compared with the Nipponbare RefSeq (IRGSP 1.0)

*: the introgression regions for each plant are listed in S6 Table.

‘: *Indica*: average number of SNPs for three modern varieties, IR64, TCS10 and TCS17. *Japonica*: average number of SNPs for three modern varieties, TNG67, TC65 and TK9.

The introgression area in chromosome 11 was the highest, and no introgression occurred in chromosome 8. The introgression region for other chromosomes ranged from 10% to 40%. Using the sum of any possible introgressed region for all seven plants, the estimated introgression for 12 chromosomes in T_1_ plants was 26.4%, about (½)^2^. Thus, the introgression might have occurred one generation before the seed was used for transformation.

### T-DNA integration and footprint analysis

Flanking sequence analysis of the M0028590 mutant indicated the T-DNA was integrated at the 1^st^ exon of an expressed gene, Os03g0762800, and the disruption of this gene did not co-segregate with the large-grain or tillering dwarf phenotypes. The T-DNA integration was homozygous in plants T_1_c and T_2_a, heterozygous in T_1_a and T_1_b, and azygous in T_2_b, T_3_a, and T_3_b.

We demonstrated high SNP frequencies for this TRIM line and suggest that such high-frequency sequence changes were likely caused by introgression. The seven T_1_ to T_3_ plants sequenced represented several types, including wild type (regular plant height and regular grain size), regular plant height and large grain, tilling dwarf and large grain as well as tillering dwarf and regular grain size. The introgression may have occurred one generation before the seed was used for transformation; thus, the T_1_, T_2_, and T_3_ plants were equivalent to the F_3_, F_4_, and F_5_ generations after the spontaneous outcrossing. The homozygous introgression regions may have decreased to 200 kb in the 2.5-Mb region of chromosome 10 for the T_2_ and T_3_ generations and to 500 kb in the 25-Mb region in the same chromosome for the T_1_ and T_2_ plants. Thus, the homozygous introgression region may have been reduced to a small region within five generations. Next-generation sequencing (NGS) provided an efficient tool to precisely define haplotypes.

Fig 3 also illustrates the footprint of how the recombination and selfing progressed, with the introgressed *indica* fragments becoming homozygous or with decreased percentage in the whole genome. In addition, at the zero generation of the introgression, chromosome 8 from pollen did not combine into the zygote, as compared with most, if not all, of chromosome 11.

### Introgression is responsible for the large-grain trait

To search for the gene responsible for the large-grain trait of the type B mutant, we used the modified MutMap+ (i.e., the modified MutMap strategy [35, 36]). We first prepared a cross between a T_2_ large-grain plant and TK9 because both TNG67 and TK9 are *japonica* rice and their SNP rates are tens of thousands, ideal for a parental line to search for markers used in the NGS mapping strategy. All F_1_ plants were wild type (i.e., regular plant height and grain size). Among the 1,000 F_2_ plants, about one quarter had large grains, which suggests a single recessive trait. We then prepared two DNA bulks from the F_2_ plants. The first consisted of 20 wild-type F_2_ plants and the other 20 large-grain F_2_ plants. We also sequenced two F_2_ large-grain plants, plants 1 and 2. The average depth and genome coverage rates are in Table 1. Fig 1 illustrates the grain size of the parental lines and the F_2_ segregated plants.

**Fig 5:**
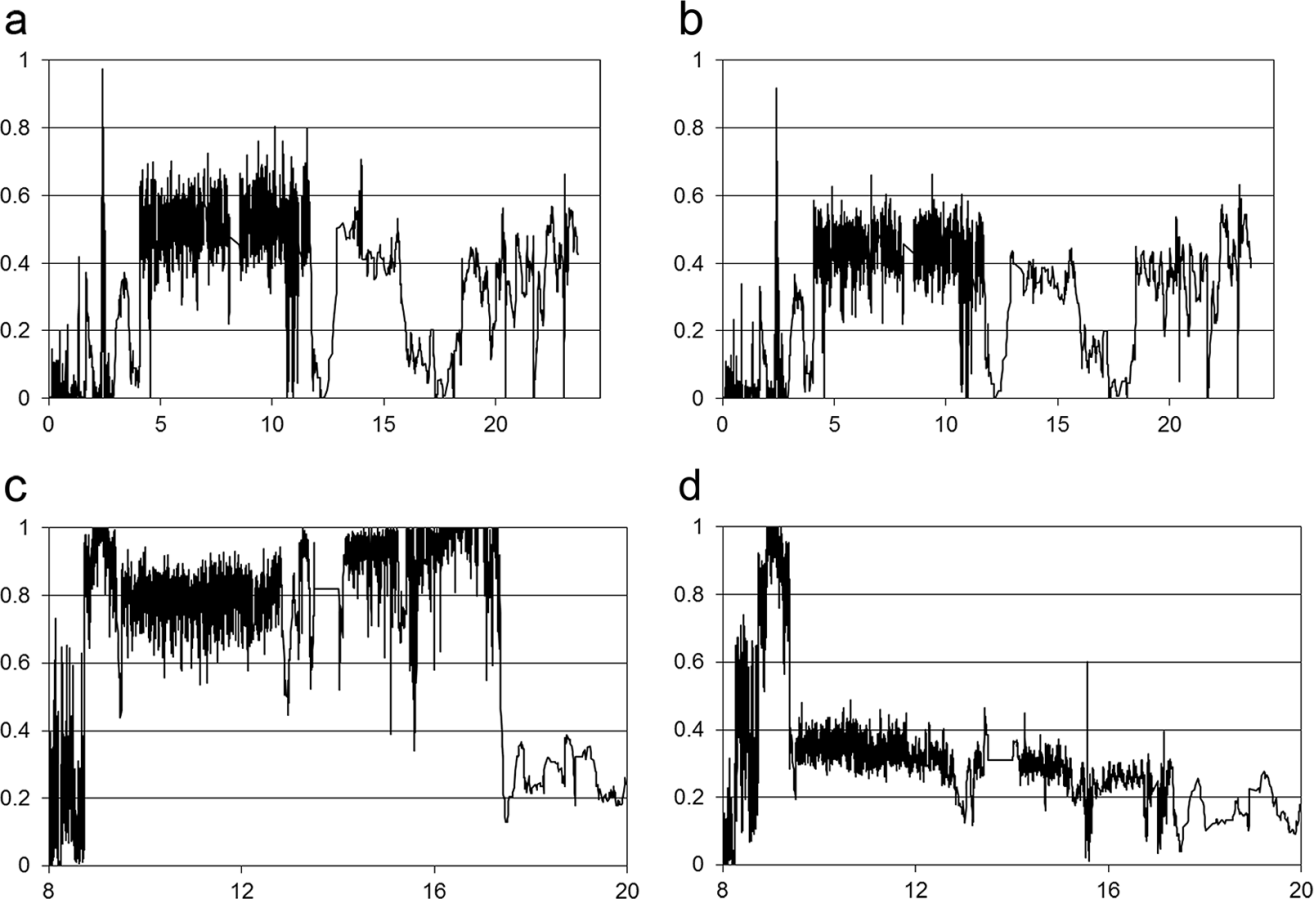
SNP frequency plots for the F_2_ DNA pools of wild-type or large-grain plants. (a) and (b) show chromosome 10 for large-grain and wild-type plants, respectively. The lines were plotted as described in Methods. The x-axis value for each averaged SNP frequency was set at a midpoint between the first and fifth SNP, and the y-axis is the position (in Mb) of the chromosome. (c) and (d) show the 8- to 20-Mb region of chromosome 3 for large-grain and wild-type plants, respectively.

Fig 5A and 5B illustrate that the SNP index (mutated SNP number/total read numbers) of both DNA pools was about 0.5 for most regions of chromosome 10, and the curves for the wild-type pool and large-grain pool show a similar pattern. This pattern was true for all other chromosomes (S3 Fig), except for the central region of chromosome 3 (Fig 5C and 5D, S3E and S3F Fig). The SNP frequency for the large-grain pool approaches one from the 14- to 17.5-MB region of chromosome 3 (Fig 5C), whereas that for the wild-type pool was about 0.4 (Fig 5D). Because of the extreme difference between the two DNA pools located at this region, the candidate gene was narrowed down to that area. After checking the SNPs and indels of the large-grain DNA pool in this region, the mutation located at C to A of a known seed-size–controlling gene *GS3* [37–39], with the exact position at 16,733,441 bp (IRGSP v.1). Sequencing data indicated that it was “C” in TNG67, TK9 and the wild-type segregants but “A” in the large-grain plants, including the parent, F_2_ pool, and the two F_2_ single plants.

*GS3* is one of the most important grain-length quantitative trait loci [37], and this C-to-A mutation occurring at the second exon of a transmembrane domain-containing locus led to an early stop of the protein and increased rice grain length [39]. A total of 180 rice accessions was used to confirm that this functional SNP was responsible for the long-grain trait [38]. None of the Taiwanese *japonica* rice varieties, including TNG67 or TK9, contain this functional SNP, as compared with some *indica* varieties, such as TCS17 or IR64. The M0028590 line contained an introgressed region from *indica* rice, as indicated previously. Among the nine plants sequenced, T_1_c (large grain), F_2_ large-grain plant 1 and F_2_ large-grain plant 2 showed homozygous introgression in the 16.7-Mb region of chromosome 3. However, for the other six plants with regular grain size, T_1_a, T_1_b, T_2_b, T_3_a and T_3_b had heterozygous introgression, and T_2_a had no introgression in the region (Fig 3). Thus, the cosegregation indicated again that homozygous introgression in the *GS3* gene region was related to the large-grain trait.

## Discussion

Using the phenomics and FST data of the TRIM population, we previously found several candidate genes responsible for the big-grain trait. However, there was no correlation between this trait and T-DNA integration for M0028590. According to the rice breeders’ record, M0028590 had three distinct phenotypes–wild type, large grains, and dwarf and high tillering number. We used seven plants, including T1, T2, and T3 plants, to reveal the sequence changes for this TRIM line and demonstrated that introgression from an *indica* variety might occur in the seed used for generation of this specific TRIM line.

### A single base change is responsible for the dwarf tillering trait

Five dwarf tillering mutants (i.e., about one-half to two-thirds of the regular plant height and three- to six-fold tiller numbers) have been reported in rice, and genes corresponding to the *d3*, *d10*, *d14*, *d17*, and *d27* mutants have been cloned [40–43]. All these genes encode proteins responsible for synthesis or regulation of strigolactone. We investigated the sequence changes in these candidate genes from the NGS data for M0028590 offspring, including the sequences of tillering dwarf plants (i.e., T_1_c, T_2_b, T_3_a and T_3_b) versus those with regular plant height and tiller number (i.e., T_1_a, T_1_b, and T_2_a) for all candidate genes (*d3*, *d10*, *d14*, *d17*, and *d27*). A homozygous SNP (from G to A) occurred at the gene Os04g0550600 specific to the 4 tillering dwarf plants. This is the *D17* gene encoding for a carotenoid cleavage oxygenase required for negative regulation of axillary bud outgrowth [42]. This SNP located at chromosome 4, position 27,570,277 bp, and led to an early stop for the 4 tillering dwarf plants. Without a functional D17 protein, the plant became high tillering with severe dwarfism. However, this SNP was not present or in heterozygous status in plants T_1_a, T_1_b, and T_2_a, with wild-type plant height and normal tiller number (Table 2).

A review paper published decades ago concluded that plant cell culture itself generated genetic variability (i.e., somaclonal variation; [44]). A similar topic studied extensively with different organisms demonstrated that such variation occurred in culture sub-clones and in regenerated plants. Somaclonal variation, resulting from a sum of genetic and epigenetic changes, might occur during callus induction, growth, *Agrobacterium* co-culture, and regeneration. Miyao et al. [45] sequenced the genomes of three rice *Tos17* mutant lines and concluded that besides integration of the retrotransposon *Tos17*, SNPs and indels were the major causes of somaclonal variation. The estimated mutation rate was 17.4 x 10^−7^ per site per regenerant. We previously sequenced the genome for four TRIM mutant lines [25], and the estimated mutation rate was 8.9–21.0 x 10^−7^ per site per diploid genome. Thus, we suggest that the SNPs occurring at *D17* were responsible for the tillering dwarf trait in the rice M0028590 line we investigated and occurred during the period of callus induction to the regeneration of T_0_ plants.

Since 2002, we have generated ~130,000 TRIM lines, numbered from the beginning of the project. We checked the phenomics data for the frequencies of large grains and large grains + tillering dwarf and found lines with a region with extremely high frequency of large grains close to M0028590; they are M0027046, M0027912, M0028590, M0029836, and M0030998. Among all lines with phenomics data, only two contained large grains + tillering dwarf: M0027912 and M0028590. We suggest that the five TRIM lines near M0028590 (included) with the large-grain phenotype came from the same intregressed seed in the tissue culture lab. In addition, the two TRIM lines with both large grains + tillering dwarf were derived from the same pieces of callus that contained the introgressed genome and a SNP in *D17*.

### Introgression occurs in the rice field

To study the sequence changes in regenerants and transformants, we prepared about 30 independent lines for statistical analysis [25]. Regenerant R, one of the regenerants produced, showed completely heterozygous SNPs across the genome. Its SNP frequency across the whole genome was 2,386,101, thousands of times higher than for any other regenerants/transformants or TRIM lines. S3 Fig illustrates the heterozygosity of all 12 chromosomes: the heterozygosity occurred across the whole genome instead of in a certain area. Gene flow may have occurred in the seed we used to regenerate the plant, with pollen from an *indica* rice variety grown nearby; that is, introgression occurred in the specific seeds used to produce the Regenerant R.

In the current study, we demonstrate two introgression cases of rice transformation or regeneration using the NGS data. Tseng et al. [46] studied the possibility of pollen dispersal in the rice field using two *japonica* varieties, TNG67 and glutinous Tainung 73 (TNG73): the outcrossing rate of TNG73 seeds was quite high at 1 m away from the pollen donor TNG67 and decreased to 0% beyond 40 m. In addition, the outcrossing rate differed in different directions. Thus, pollen dispersal in the rice field occurs and may have caused the introgression.

Most other rice-consuming countries grow only *japonica* or *indica* varieties; however, Taiwan grows and consumes both types. Thus, breeders in research institutes always grow both types side by side. Because the SNP rates are very high between *japonica* and *indica* rice, we found introgression in the materials we used in the current study. However, the SNP rate among *japonica* or among *indica* was lower, and thus the introgression is not easy to detect even if it occurs. In addition, to reveal sequence changes, we used NGS, which is very sensitive as compared with phenotype observations or use of molecular markers for plants grown in field conditions.

To eliminate the possibility of introgression occurring in the field, we recommend (1) covering with a bag to prevent pollen cross-contamination during the heading time, (2) planting more border lines in all directions, and (3) growing important lines in the upwind field.

## Conclusions

The relationship between genotype and phenotype for the rice insertional mutant population is very low. In our study of the M0028590 mutant line and Regenerant R, we demonstrate that introgression from *indica* rice occurred from the seeds used for callus induction. We show how the introgression area, including homozygous and heterozygous areas, changed in different generations after the hybridization in M0028590. T-DNA integration was not associated with the mutated phenotypes, and we illustrate the reasons for the large-grain (type B trait) and tillering dwarf (type C trait) phenotypes. Thus, in addition to the known sequence changes such as T-DNA integration, SNP, insertion, deletion, chromosome rearrangement and doubling, spontaneous outcrossing occurring in the rice field may also explain some mutated traits in a tagged mutant population.

## Supporting Information

S1 Fig Phylogenic tree of M0028590 and other *japonica* and *indica* rice varieties. The tree was constructed with SNP data from 7 M0028590 offspring, 5 *indica*, 2 *Aus*, 7 *japonica* rice, 1 regenerant, and 4 TRIM mutant lines by using SNPhylo [34]. The whole rice genome was used for analysis. The accession number and type of the 25 materials are in Table 1 and S3 Table.
(TIF)

S2 Fig Single nucleotide polymorphism (SNP) plot of several rice lines. The x-axis is the position in chromosome 1 and the y-axis the SNP density, with the unit SNP/2 Mb. The line and code are as indicated. Four mutant lines were all generated from TNG67. M0028590T_1_b and M0028590T_3_a are the offspring of M0028590. M0048349 and M0053677 are other two TRIM lines as a control. TaichungSen 17 (TCS17) is a local *indica* rice variety for comparison.
(TIF)

S3 Fig SNP frequency plots for the F_2_ DNA pools of large-grain and wild-type plants. The x-axis is the 12 chromosomes and the y-axis is the SNP index (mutated SNP number/total read numbers). (a) and (b) show chromosome 1; (c) and (d) chromosome 2; (e) and (f) chromosome 3; (g) and (h) chromosome 4; (i) and (j) chromosome 5; (k) and (l) chromosome 6; (m) and (n) chromosome 7; (o) and (p) chromosome 8; (q) and (r) chromosome 9; (s) and (t) chromosome 11; and (u) and (v) chromosome 12 for large-grain and wild-type plants, respectively. The lines were obtained by averaging SNP frequencies from a moving window of 20 consecutive SNPs and shifting the window one SNP at a time. The y-axis value for each averaged SNP frequency was set at a midpoint between the first and fifth SNP.
(PDF)

S4 Fig Heterozygous SNP plot for Regenerant R. The x-axis is the position on the chromosome and y-axis is the heterozygous SNP counts. Colored dots represent SNPs for Regenerant R. Red horizontal line is the mean of 100 chosen *indica* and green line is the mean of 100 chosen *japonica* from the 3K rice project (S7 Table).
(PDF)

S1 Table Accession numbers from variant call format (VCF) files generated. All VCF information is available in the European Variation Archive (http://www.ebi.ac.uk/eva/). The project accession numbers and plants in each accession number are listed.
(DOCX)

S2 Table Number of single nucleotide polymorphisms (SNPs) for *indica* or *japonica* varieties vs Nipponbare. The average number of SNPs among 100 accessions of *indica* or *japonica* rice from the 3000 rice genome project and varieties usually grown in breeders’ fields in Taiwan.
(DOCX)

S3 Table Information on rice sequences used for phylogenetic analysis. TRIM lines, *indica*, *japonica*, and *Aus* rice were used.
(DOCX)

S4 Table The 18-Mb region used for the phylogenetic analysis. Regions where no introgression occurred in T_2_a plants but introgression occurred in the other 6 M0028590 offspring.
(DOCX)

S5 Table Characteristics of sequence changes for the homozygous introgression region in T_1_C plant. SnpEff was used to run the analysis [47].
(XLSX)

S6 Table Introgression regions of M0028590 offspring compared with TNG67. (DOCX)

S7 Table Accessions used for calculating SNPs against Nipponbare in the 3000 rice genome project. (DOCX)
