# Supplementary figures and images for: Lack of Genotype and Phenotype Correlation in a Rice T-DNA Tagged Line Is Likely Caused by Introgression in the Seed Source
Source: PLoS One. 2016 May 17;11(5):e0155768. doi: 10.1371/journal.pone.0155768 (PMC4871347; doi:10.1371/journal.pone.0155768)

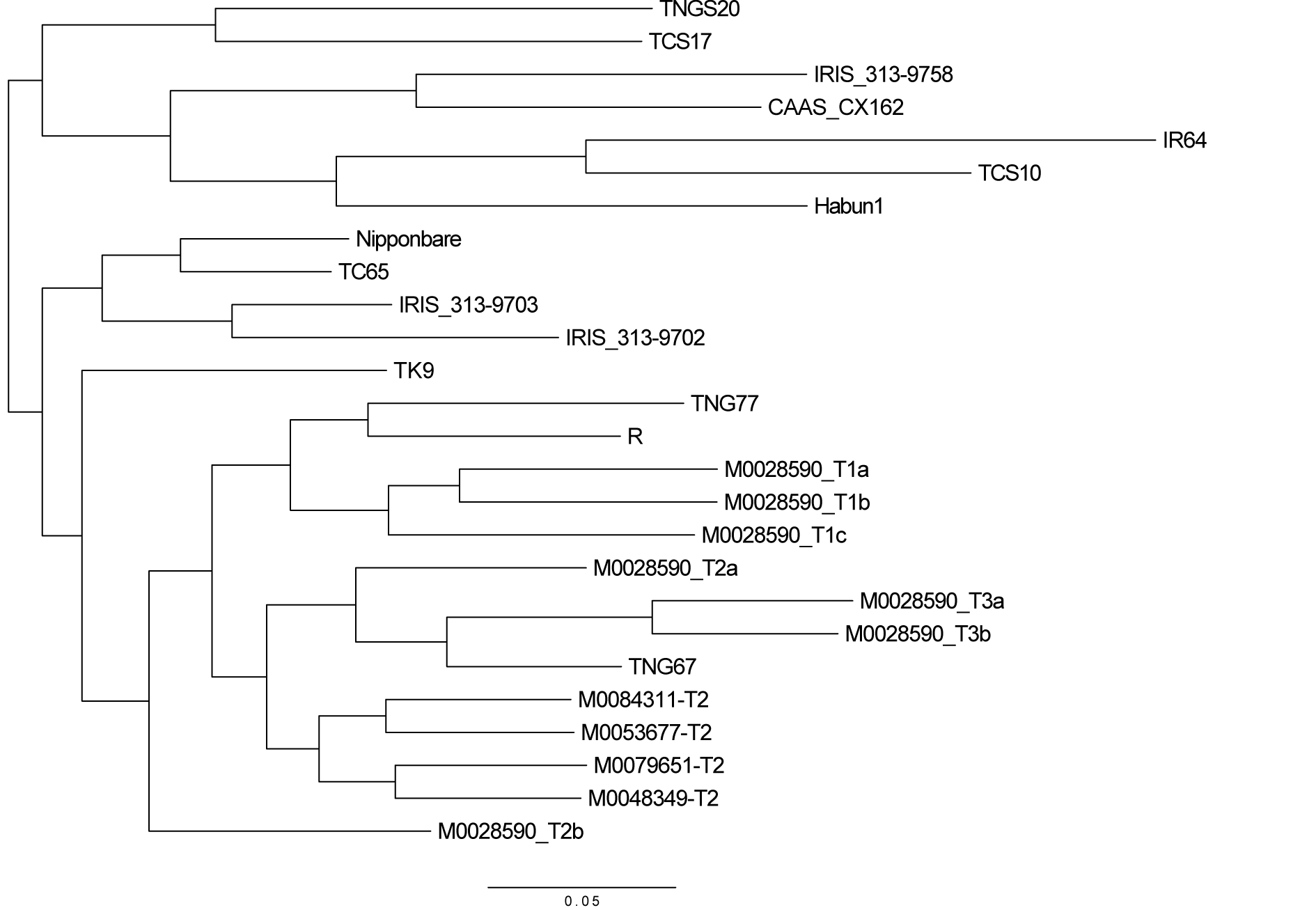

Supplement: S1 Fig — The tree was constructed with SNP data from 7 M0028590 offspring, 5 indica, 2 Aus, 7 japonica rice, 1 regenerant, and 4 TRIM mutant lines by using SNPhylo [34]. The whole rice genome was used for analysis. The accession number and type of the 25 materials are in Table 1 and S3 Table. (TIF) [file pone.0155768.s001.tif]

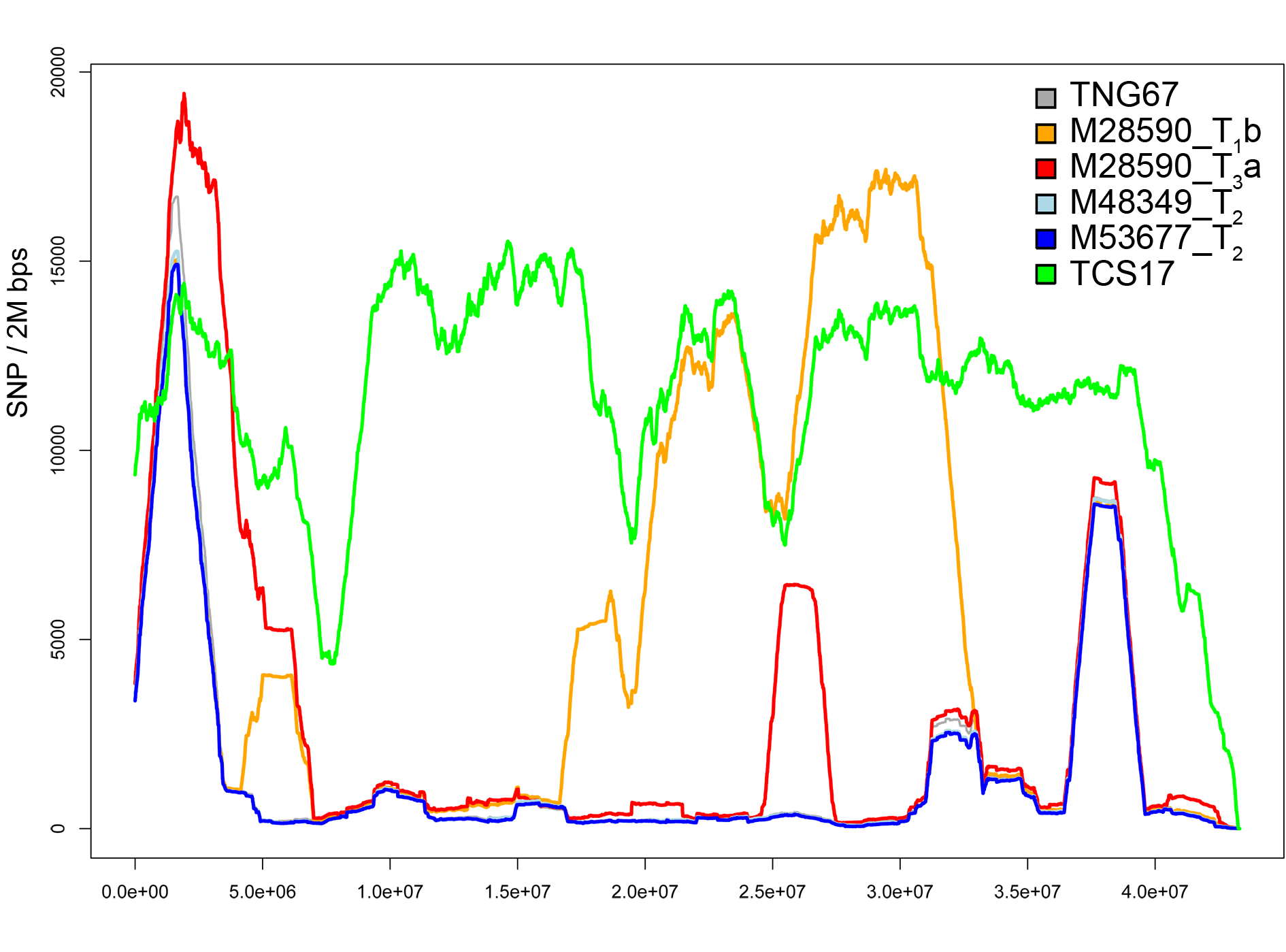

Supplement: S2 Fig — The x-axis is the position in chromosome 1 and the y-axis the SNP density, with the unit SNP/2 Mb. The line and code are as indicated. Four mutant lines were all generated from TNG67. M0028590T1b and M0028590T3a are the offspring of M0028590. M0048349 and M0053677 are other two TRIM lines as a control. TaichungSen 17 (TCS17) is a local indica rice variety for comparison. (TIF) [file pone.0155768.s002.tif]

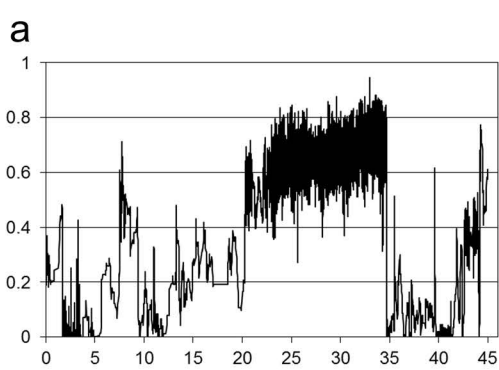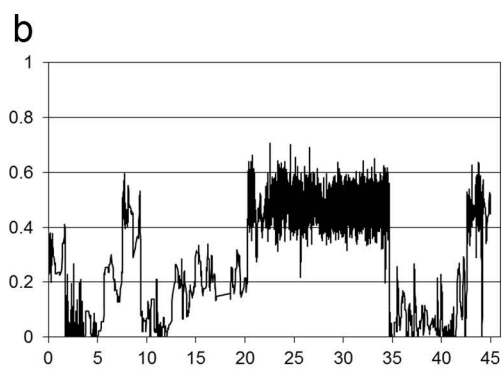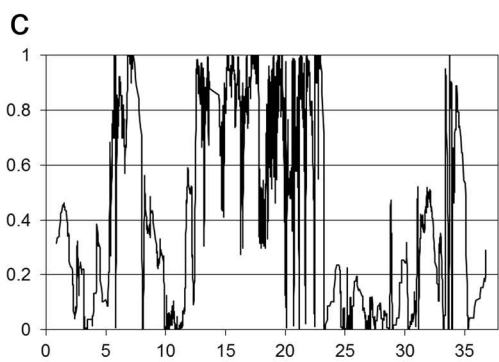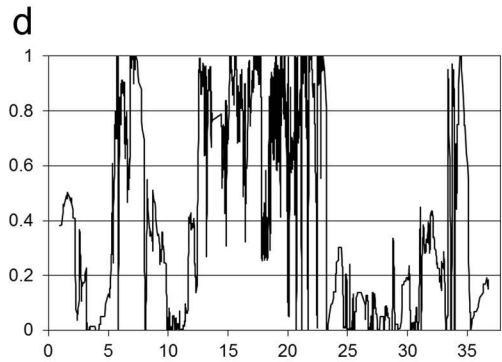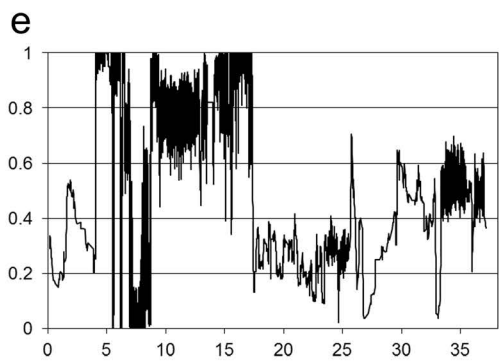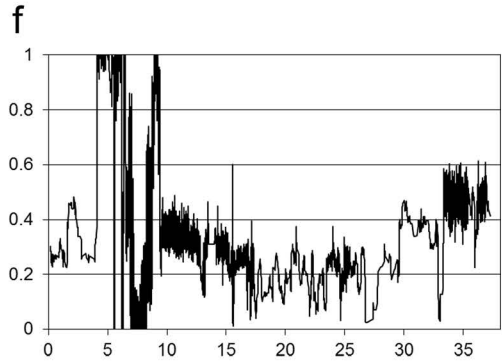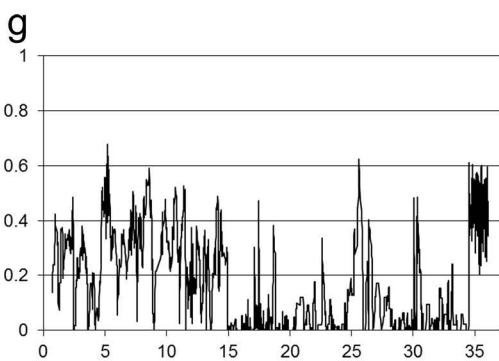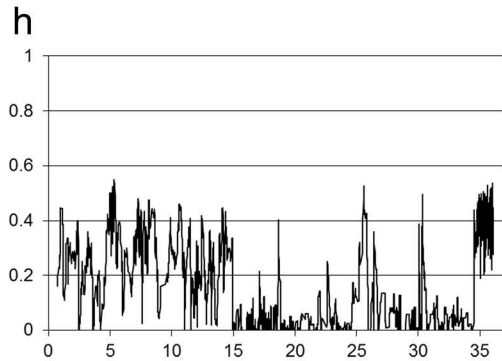

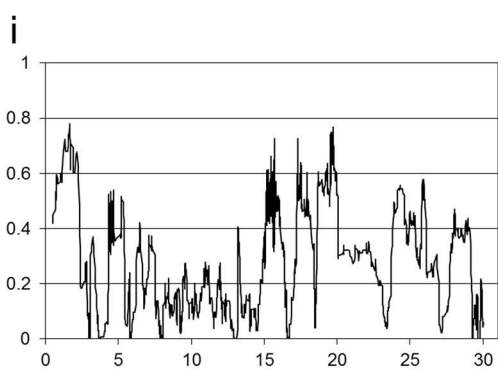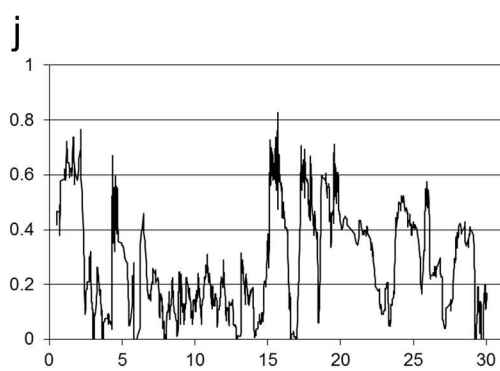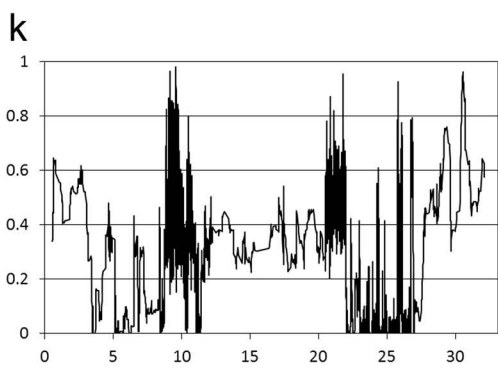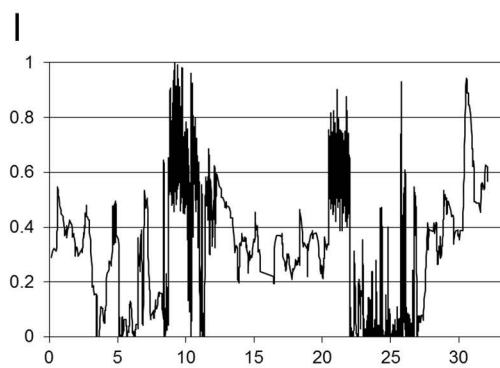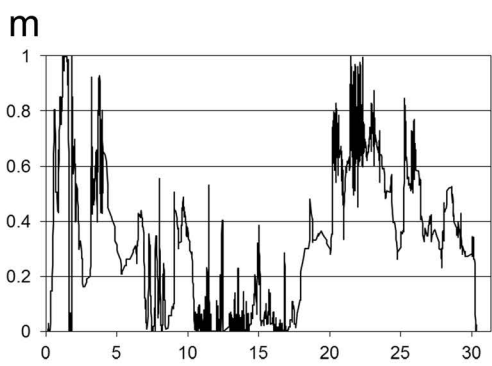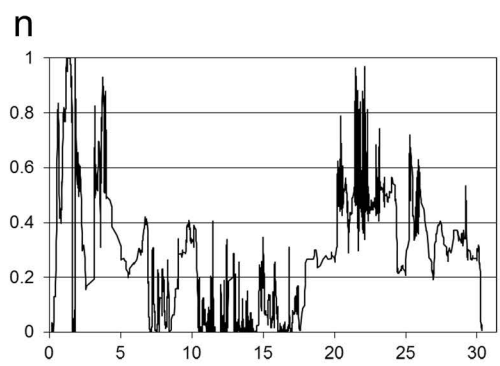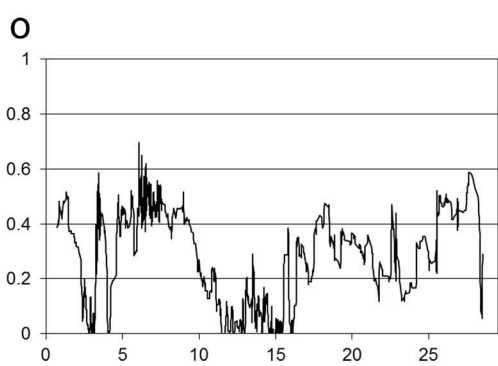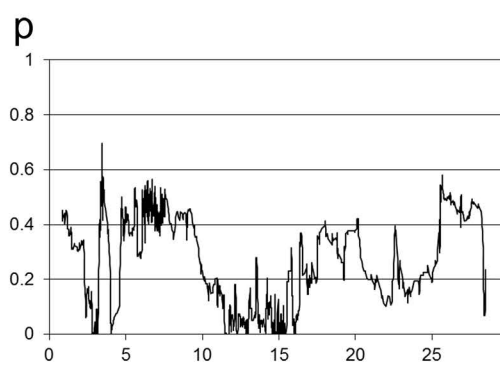

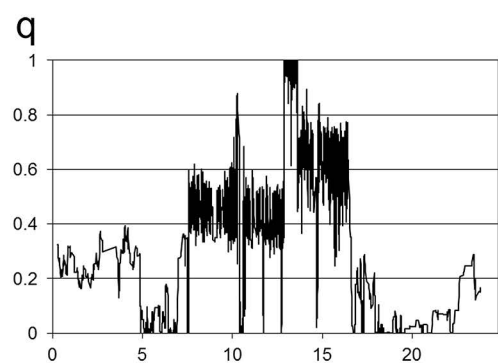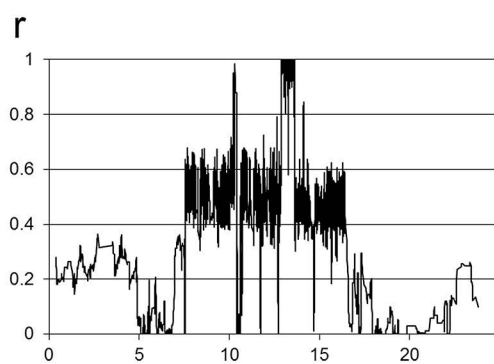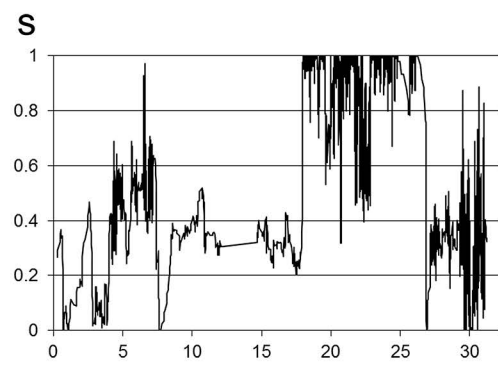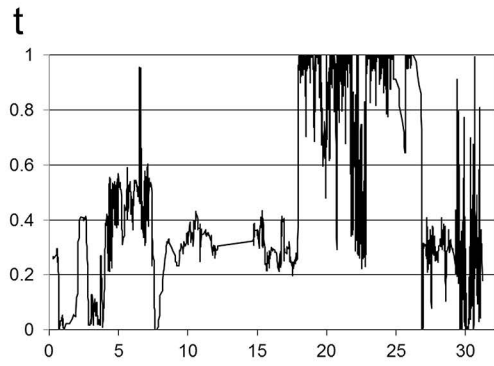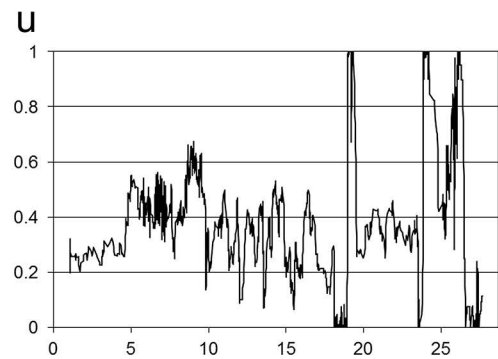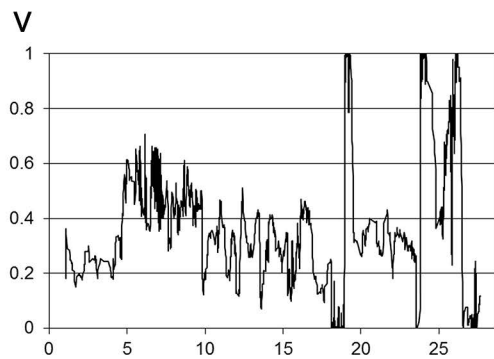

Supplement: S3 Fig — The x-axis is the 12 chromosomes and the y-axis is the SNP index (mutated SNP number/total read numbers). (a) and (b) show chromosome 1; (c) and (d) chromosome 2; (e) and (f) chromosome 3; (g) and (h) chromosome 4; (i) and (j) chromosome 5; (k) and (l) chromosome 6; (m) and (n) chromosome 7; (o) and (p) chromosome 8; (q) and (r) chromosome 9; (s) and (t) chromosome 11; and (u) and (v) chromosome 12 for large-grain and wild-type plants, respectively. The lines were obtained by averaging SNP frequencies from a moving window of 20 consecutive SNPs and shifting the window one SNP at a time. The y-axis value for each averaged SNP frequency was set at a midpoint between the first and fifth SNP. (PDF) [file pone.0155768.s003.pdf]

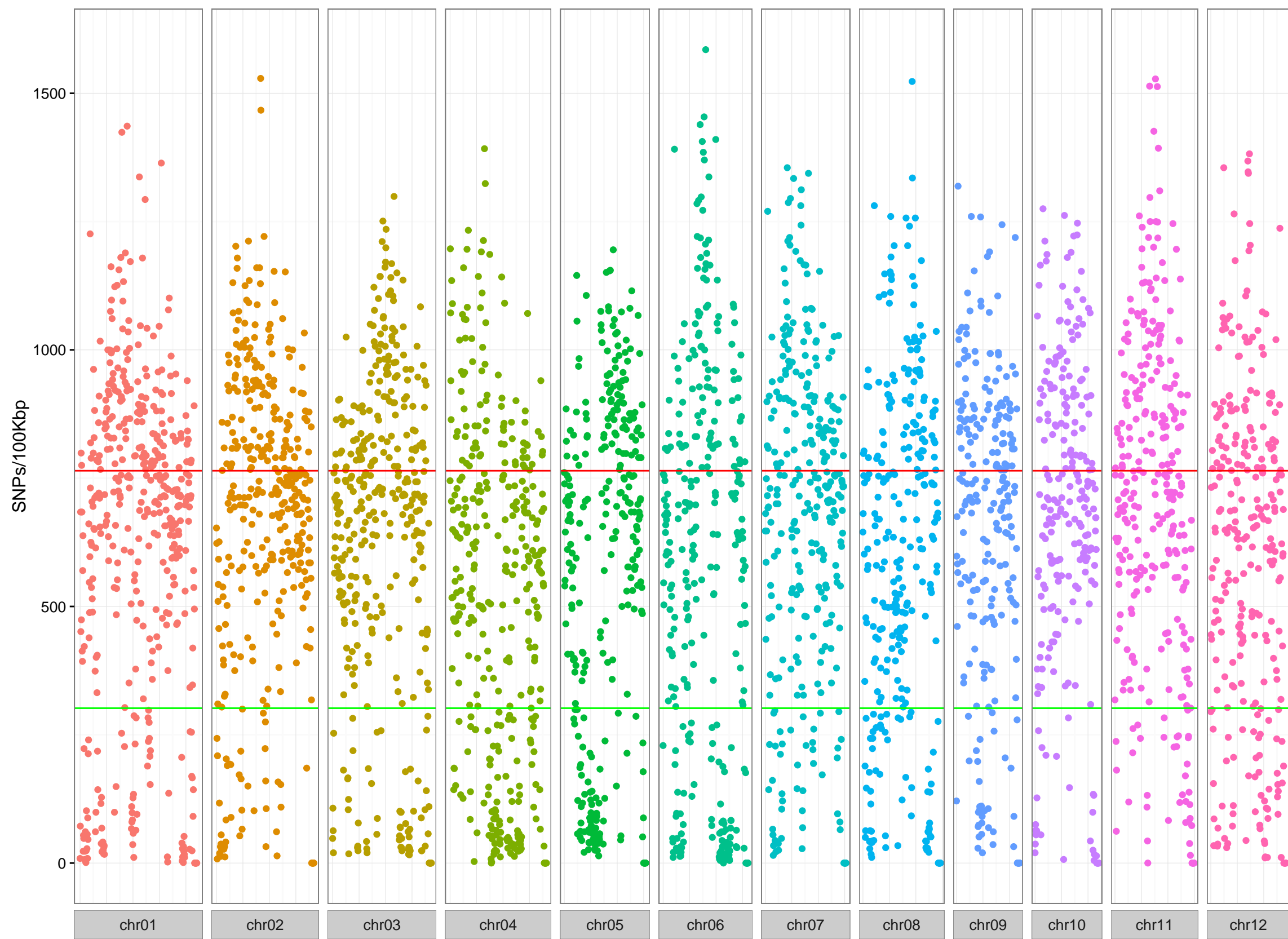

Supplement: S4 Fig — The x-axis is the position on the chromosome and y-axis is the heterozygous SNP counts. Colored dots represent SNPs for Regenerant R. Red horizontal line is the mean of 100 chosen indica and green line is the mean of 100 chosen japonica from the 3K rice project (S7 Table). (PDF) [file pone.0155768.s004.pdf]
