# Supplementary material for: Lack of Genotype and Phenotype Correlation in a Rice T-DNA Tagged Line Is Likely Caused by Introgression in the Seed Source
Source: PLoS One. 2016 May 17;11(5):e0155768. doi: 10.1371/journal.pone.0155768 (PMC4871347; doi:10.1371/journal.pone.0155768)
Supplement: S1 Table — All VCF information is available in the European Variation Archive (http://www.ebi.ac.uk/eva/). The project accession numbers and plants in each accession number are listed. (DOCX) [file pone.0155768.s005.docx]

**S1 Table. Accession numbers in VCF files generated.**

| Accession Name | Analysis Alias | Project accession number |
| --- | --- | --- |
| Taichung 65 (TC65) | Var_TC65 | PRJEB12769 |
| Taichung Sen 10 (TCS10) | Var_TCS10 | PRJEB12769 |
| Taichung Sen 17 (TCS17) | Var_TCS17 | PRJEB12769 |
| Taikeng 9 (TK9) | Var_TK9 | PRJEB12769 |
| Tainung 67 | Var_TNG67 | PRJEB12769 |
| IR64 | Var_IR64_HS | PRJEB12770 |
| Regenerant R | Var_R | PRJEB12770 |
| M0028590 T_1_a | Var_M28590_T1a | PRJEB12771 |
| M0028590 T_1_b | Var_M28590_T1b | PRJEB12771 |
| M0028590 T_1_c | Var_M28590_T1c | PRJEB12771 |
| M0028590 T_2_a | Var_M28590_T2a | PRJEB12771 |
| M0028590 T_2_b | Var_M28590_T2b | PRJEB12771 |
| M0028590 T_3_a | Var_M28590_T3a | PRJEB12771 |
| M0028590 T_3_b | Var_M28590_T3b | PRJEB12771 |
| F_2_ big-seed pool | Var_TallTk9F2_M | PRJEB12771 |
| F_2_ big-seed plant 1 | Var_TallTk9F2_p1 | PRJEB12771 |
| F_2_ big-seed plant 2 | Var_TallTk9F2_p2 | PRJEB12771 |
| F_2_ wild-type pool | Var_TallTk9F2_W | PRJEB12771 |

All information is available in the European Variation Archive (http://www.ebi.ac.uk/eva/). The project accession numbers and plants in each accession are listed.
