# Supplementary material for: Lack of Genotype and Phenotype Correlation in a Rice T-DNA Tagged Line Is Likely Caused by Introgression in the Seed Source
Source: PLoS One. 2016 May 17;11(5):e0155768. doi: 10.1371/journal.pone.0155768 (PMC4871347; doi:10.1371/journal.pone.0155768)
Supplement: S2 Table — The average number of SNPs among 100 accessions of indica or japonica rice from the 3000 rice genome project and varieties usually grown in breeders’ fields in Taiwan. (DOCX) [file pone.0155768.s006.docx]

**S2 Table. Number of single nucleotide polymorphisms (SNPs) for *indica* or *japonica* varieties vs Nipponbare.** The average number of SNPs among 100 accessions of *indica* or *japonica* rice from the 3000 rice genome project and varieties usually grown in breeders’ fields in Taiwan.

|  | **100 accessions**  **from 3K project^a^** | **Varieties usually grown**  **in breeders’ fields in Taiwan^b^** |
| --- | --- | --- |
| ***Indica*** | 2,852,440± 410,550 | 2,549,930± 543,750 |
| ***Japonica*** | 1,125,990± 580,440 | 188,520± 83,450 |

^a^The first 100 *indica* and first 100 *japonica* accessions for the IRIS_313-x series. The accessions are in S5 Table.

^b^*The indica* rice varieties are TCS10, TCS17, and IR64. The *japonica* rice varieties are TNG67, TC65, and TK9.
