# Supplementary material for: Lack of Genotype and Phenotype Correlation in a Rice T-DNA Tagged Line Is Likely Caused by Introgression in the Seed Source
Source: PLoS One. 2016 May 17;11(5):e0155768. doi: 10.1371/journal.pone.0155768 (PMC4871347; doi:10.1371/journal.pone.0155768)
Supplement: S3 Table — TRIM lines, indica, japonica, and Aus rice were used. (DOCX) [file pone.0155768.s007.docx]

**S3 Table. information on rice sequences used for phylogenetic analysis.** TRIM lines, *indica, japonica*, and *Aus* rice were used.

|  | **Read type^a^** | **Estimated average**  **depth^b^** | **Genome coverage**  **ratio** | **Rice type** | **Accession number** |
| --- | --- | --- | --- | --- | --- |
| **M0048349-T_2_** | 101PE | 14.82 | 98.07 % | TRIM | SRR2767710 |
| **M0053677-T_2_** | 101PE | 14.51 | 97.37 % | TRIM | SRR2767711 |
| **M0079651-T_2_** | 101PE | 15.05 | 98.11 % | TRIM | SRR2767712 |
| **M0084311-T_2_** | 101PE | 15.07 | 97.52 % | TRIM | SRR2767713 |
| **Habun1** | 126PE | 16.87 | 89.91 % | *Aus* | SRR2106586 |
| **TNGS20** | 75PE | 7.16 | 85.01 % | *Indica* | SRR3405165 |
| **TNG77** | 126PE | 18.52 | 96.74 % | *Indica* | SRR3405166 |
| **IRIS_313-9702*** | 83PE | 13.94 | 96.66 % | *Japonica* | ERS468332 |
| **IRIS_313-9703*** | 83PE | 7.06 | 94.57 % | *Japonica* | ERS467887 |
| **IRIS_313-9758*** | 83PE | 13.49 | 91.19 % | *Aus* | ERS468113 |
| **CAAS_CX162*** | 83PE | 19.28 | 90.09 % | *Indica* | ERS470527 |

*****IRIS_313-9702, TAICHUNG 179; IRIS_313-9703, CHIANAN 8; IRIS_313-9758, I-KUNG-PAO; CAAS_CX162, Taichung native 1.
