# Supplementary material for: Lack of Genotype and Phenotype Correlation in a Rice T-DNA Tagged Line Is Likely Caused by Introgression in the Seed Source
Source: PLoS One. 2016 May 17;11(5):e0155768. doi: 10.1371/journal.pone.0155768 (PMC4871347; doi:10.1371/journal.pone.0155768)
Supplement: S4 Table — Regions where no introgression occurred in T2a plants but introgression occurred in the other 6 M0028590 offspring. (DOCX) [file pone.0155768.s008.docx]

**S4 Table. The 18-Mb region used for the phylogenetic analysis.** Regions where no introgression occurred in T_2_a plants but introgression occurred in the other 6 M0028590 offspring.

|  | Start..end | T_1_a | T_1_b | T_1_c | T_2_a | T_2_b | T_3_a | T_3_b |
| --- | --- | --- | --- | --- | --- | --- | --- | --- |
| chr02 | 22110000..22280000 | Hm | He | Hm | - | Hm | Hm | Hm |
| chr03 | 12160000..15514527 | He | He | Hm | - | He | He | Hm |
| chr03 | 15515115..15920000 | He | He | Hm | - | He | He | He |
| chr07 | 19510000..20000000 | He | He | Hm | - | Hm | Hm | Hm |
| chr07 | 26370000..28060000 | Hm | He | Hm | - | Hm | Hm | Hm |
| chr11 | 4360000..7530000 | Hm | Hm | He | - | Hm | Hm | He |
| chr11 | 8950000..14691164 | Hm | Hm | He | - | Hm | Hm | He |
| chr11 | 14691553..17870000 | Hm | Hm | He | - | He | He | He |
