# Supplementary material for: Lack of Genotype and Phenotype Correlation in a Rice T-DNA Tagged Line Is Likely Caused by Introgression in the Seed Source
Source: PLoS One. 2016 May 17;11(5):e0155768. doi: 10.1371/journal.pone.0155768 (PMC4871347; doi:10.1371/journal.pone.0155768)
Supplement: S6 Table — (DOCX) [file pone.0155768.s010.docx]

**S6 Table. Introgression regions of M0028590 offspring compared with TNG67.**

|  | Start..end | T_1_a | T_1_b | T_1_c | T_2_a | T_2_b | T_3_a | T_3_b |
| --- | --- | --- | --- | --- | --- | --- | --- | --- |
| chr01 | 3320000..7010000 | - | - | - | - | Hm | Hm | Hm |
| chr01 | 18650000..24870000 | He | He | He | - | - | - | - |
| chr01 | 24870000..26440000 | He | He | Hm | He | - | - | - |
| chr01 | 26440000..27482554 | He | He | Hm | He | Hm | Hm | Hm |
| chr01 | 27482554..27482856 | He | Hm | Hm | He | - | Hm | Hm |
| chr01 | 27482856..27490000 | He | Hm | Hm | He | He | Hm | Hm |
| chr01 | 27490000..29920000 | He | Hm | Hm | He | He | - | - |
| chr01 | 29920000..33000000 | He | Hm | He | He | - | - | - |
| chr02 | 17060000..18640000 | Hm | He | Hm | He | He | He | He |
| chr02 | 18640000..18808439 | Hm | He | Hm | He | - | - | - |
| chr02 | 18808439..19120050 | Hm | He | Hm | He | He | He | He |
| chr02 | 19120050..19120080 | Hm | He | Hm | He | - | - | - |
| chr02 | 19120080..22110000 | Hm | He | Hm | He | Hm | Hm | Hm |
| chr02 | 22110000..22280000 | Hm | He | Hm | - | Hm | Hm | Hm |
| chr02 | 32870000..33930000 | He | He | - | - | - | - | - |
| chr02 | 33930000..34690000 | He | He | - | - | Hm | Hm | Hm |
| chr02 | 34690000..35430000 | He | He | - | He | Hm | Hm | Hm |
| chr02 | 35430000..35470000 | He | He | - | He | - | - | - |
| chr02 | 35470000..35630000 | - | - | - | He | - | - | - |
| chr03 | 6860000..7504194 | Hm | Hm | He | - | He | - | He |
| chr03 | 7504194..7504625 | Hm | - | He | - | He | - | He |
| chr03 | 7504625..10637937 | Hm | He | He | - | He | - | He |
| chr03 | 10637937..10638500 | - | He | He | - | He | - | He |
| chr03 | 10638500..10696732 | He | He | He | - | He | - | He |
| chr03 | 10696732..10696804 | He | He | He | - | He | - | - |
| chr03 | 10696804..11444758 | He | He | He | - | He | - | Hm |
| chr03 | 11444758..11444811 | He | He | - | - | He | - | Hm |
| chr03 | 11444811..12160000 | He | He | Hm | - | He | - | Hm |
| chr03 | 12160000..15514527 | He | He | Hm | - | He | He | Hm |
| chr03 | 15514527..15515115 | He | He | Hm | - | He | He | - |
| chr03 | 15515115..15920000 | He | He | Hm | - | He | He | He |
| chr03 | 15920000..16670000 | He | He | Hm | - | - | - | - |
| chr03 | 22610000..23970000 | He | - | Hm | - | - | - | - |
| chr03 | 23970000..25170000 | He | - | Hm | He | - | - | - |
| chr03 | 32380000..32430000 | - | - | - | He | - | - | - |
| chr03 | 32430000..36070000 | He | He | Hm | He | - | - | - |
| chr03 | 36070000..36420000 | - | - | - | He | - | - | - |
| chr04 | 0..2490000 | He | - | - | - | - | - | - |
| chr04 | 6860000..7060000 | - | - | - | - | He | - | - |
| chr04 | 33990000..34972179 | - | - | Hm | - | Hm | Hm | Hm |
| chr04 | 34972179..34973044 | - | - | - | - | Hm | Hm | Hm |
| chr04 | 34973044..35502694 | - | - | He | - | Hm | Hm | Hm |
| chr04 | 35502694..35510000 | - | - | - | - | Hm | Hm | Hm |
| chr05 | 4300000..5390000 | He | He | Hm | Hm | - | - | - |
| chr05 | 5390000..5410000 | - | - | - | Hm | - | - | - |
| chr05 | 16880000..18746436 | - | He | Hm | Hm | - | - | - |
| chr05 | 18746436..18746765 | - | He | Hm | - | - | - | - |
| chr05 | 18746765..18850000 | - | He | Hm | He | - | - | - |
| chr06 | 4630000..5110418 | He | He | - | Hm | - | - | - |
| chr06 | 5110418..5112855 | He | He | - | - | - | - | - |
| chr06 | 5112855..5240000 | He | He | - | Hm | - | - | - |
| chr06 | 5240000..5590000 | He | He | - | - | - | - | - |
| chr06 | 5590000..7530000 | He | He | - | Hm | - | - | - |
| chr06 | 7530000..8370000 | - | He | - | Hm | - | - | - |
| chr06 | 8370000..9500000 | - | He | He | Hm | - | - | - |
| chr06 | 25050000..25290000 | - | - | - | - | He | He | He |
| chr07 | 3100000..3106467 | - | Hm | Hm | - | - | - | - |
| chr07 | 3106467..3641577 | - | Hm | Hm | He | - | - | - |
| chr07 | 3641577..3650000 | - | Hm | Hm | - | - | - | - |
| chr07 | 11430000..11460000 | He | He | He | He | - | - | - |
| chr07 | 19510000..20000000 | He | He | Hm | - | Hm | Hm | Hm |
| chr07 | 20000000..20940000 | - | - | - | - | Hm | Hm | Hm |
| chr07 | 24860000..26370000 | - | - | - | - | Hm | Hm | Hm |
| chr07 | 26370000..28060000 | Hm | He | Hm | - | Hm | Hm | Hm |
| chr07 | 28060000..28110000 | - | - | - | - | Hm | Hm | Hm |
| chr07 | 28110000..28160058 | - | - | - | He | Hm | Hm | Hm |
| chr07 | 28160058..28233229 | - | - | - | He | - | Hm | Hm |
| chr07 | 28233229..28350000 | - | - | - | He | He | Hm | Hm |
| chr07 | 28350000..28570000 | He | He | He | He | He | Hm | Hm |
| chr07 | 28570000..28620000 | - | - | - | He | - | - | - |
| chr09 | 12200000..14580000 | Hm | - | Hm | Hm | He | He | - |
| chr09 | 14580000..14585701 | Hm | - | Hm | Hm | He | He | Hm |
| chr09 | 14585701..14586596 | Hm | - | Hm | Hm | - | - | Hm |
| chr09 | 14586596..14920000 | Hm | - | Hm | Hm | Hm | Hm | Hm |
| chr09 | 14920000..15730000 | Hm | He | Hm | Hm | Hm | Hm | Hm |
| chr10 | 3100000..3570000 | - | - | - | - | He | He | He |
| chr10 | 4220000..11290000 | - | Hm | Hm | - | Hm | Hm | Hm |
| chr10 | 22580000..22790000 | Hm | Hm | Hm | Hm | - | - | - |
| chr10 | 22790000..23080000 | Hm | Hm | Hm | - | - | - | - |
| chr11 | 0..2690000 | - | - | - | He | - | - | - |
| chr11 | 4350000..4360000 | Hm | Hm | He | - | - | - | - |
| chr11 | 4360000..7530000 | Hm | Hm | He | - | Hm | Hm | He |
| chr11 | 7530000..8950000 | - | - | - | - | Hm | Hm | He |
| chr11 | 8950000..14691164 | Hm | Hm | He | - | Hm | Hm | He |
| chr11 | 14691164..14691553 | Hm | Hm | He | - | - | - | He |
| chr11 | 14691553..17870000 | Hm | Hm | He | - | He | He | He |
| chr11 | 24720000..24860000 | - | - | - | He | - | - | - |
| chr11 | 24860000..24940000 | - | He | He | He | - | - | - |
| chr11 | 24940000..25610000 | - | He | He | He | He | - | Hm |
| chr11 | 25610000..26040000 | - | He | He | He | - | - | - |
| chr11 | 26040000..26340000 | - | He | He | He | He | He | He |
| chr11 | 26340000..26350000 | - | He | - | - | He | He | He |
| chr11 | 26350000..26380000 | - | - | - | - | He | He | He |
| chr11 | 27110000..27420000 | - | - | - | - | He | Hm | He |
| chr11 | 27420000..27780000 | - | - | - | - | He | - | He |
| chr12 | 0..4550000 | - | Hm | - | He | - | - | - |
| chr12 | 4550000..5920000 | - | - | - | He | - | - | - |

Hm: Homozygous region, He: Heterozygous region
