# Supplementary material for: Lack of Genotype and Phenotype Correlation in a Rice T-DNA Tagged Line Is Likely Caused by Introgression in the Seed Source
Source: PLoS One. 2016 May 17;11(5):e0155768. doi: 10.1371/journal.pone.0155768 (PMC4871347; doi:10.1371/journal.pone.0155768)
Supplement: S7 Table — (DOCX) [file pone.0155768.s011.docx]

**S7 Table. Accessions used for calculating SNPs against Nipponbare in the 3000 rice genome project.**

***Indica*:**IRIS_313-10000 ~ IRIS_313-10560

IRIS_313-10000, IRIS_313-10001, IRIS_313-10002, IRIS_313-10007, IRIS_313-10020, IRIS_313-10040, IRIS_313-10075, IRIS_313-10103, IRIS_313-10129, IRIS_313-10134, IRIS_313-10147, IRIS_313-10148, IRIS_313-10150, IRIS_313-10151, IRIS_313-10154, IRIS_313-10167, IRIS_313-10168, IRIS_313-10170, IRIS_313-10171, IRIS_313-10177, IRIS_313-10178, IRIS_313-10179, IRIS_313-10189, IRIS_313-10191, IRIS_313-10196, IRIS_313-10211, IRIS_313-10221, IRIS_313-10224, IRIS_313-10226, IRIS_313-10234, IRIS_313-10235, IRIS_313-10237, IRIS_313-10238, IRIS_313-10239, IRIS_313-10294, IRIS_313-10295, IRIS_313-10298, IRIS_313-10332, IRIS_313-10333, IRIS_313-10334, IRIS_313-10336, IRIS_313-10337, IRIS_313-10340, IRIS_313-10341, IRIS_313-10348, IRIS_313-10349, IRIS_313-10353, IRIS_313-10355, IRIS_313-10357, IRIS_313-10360, IRIS_313-10361, IRIS_313-10366, IRIS_313-10371, IRIS_313-10374, IRIS_313-10375, IRIS_313-10385, IRIS_313-10392, IRIS_313-10394, IRIS_313-10396, IRIS_313-10404, IRIS_313-10412, IRIS_313-10417, IRIS_313-10428, IRIS_313-10433, IRIS_313-10441, IRIS_313-10448, IRIS_313-10449, IRIS_313-10450, IRIS_313-10452, IRIS_313-10458, IRIS_313-10476, IRIS_313-10477, IRIS_313-10480, IRIS_313-10497, IRIS_313-10502, IRIS_313-10503, IRIS_313-10504, IRIS_313-10506, IRIS_313-10510, IRIS_313-10511, IRIS_313-10514, IRIS_313-10515, IRIS_313-10517, IRIS_313-10518, IRIS_313-10519, IRIS_313-10520, IRIS_313-10523, IRIS_313-10525, IRIS_313-10526, IRIS_313-10527, IRIS_313-10539, IRIS_313-10542, IRIS_313-10544, IRIS_313-10547, IRIS_313-10550, IRIS_313-10554, IRIS_313-10555, IRIS_313-10556, IRIS_313-10557, IRIS_313-10560

***Japonica*:** IRIS_313-10056 ~ IRIS_313-10800

IRIS_313-10056, IRIS_313-10057, IRIS_313-10059, IRIS_313-10065, IRIS_313-10067, IRIS_313-10071, IRIS_313-10073, IRIS_313-10074, IRIS_313-10076, IRIS_313-10077, IRIS_313-10078, IRIS_313-10079, IRIS_313-10080, IRIS_313-10082, IRIS_313-10092, IRIS_313-10093, IRIS_313-10094, IRIS_313-10096, IRIS_313-10097, IRIS_313-10124, IRIS_313-10152, IRIS_313-10176, IRIS_313-10190, IRIS_313-10228, IRIS_313-10359, IRIS_313-10373, IRIS_313-10379, IRIS_313-10380, IRIS_313-10423, IRIS_313-10429, IRIS_313-10430, IRIS_313-10437, IRIS_313-10440, IRIS_313-10453, IRIS_313-10459, IRIS_313-10469, IRIS_313-10485, IRIS_313-10489, IRIS_313-10507, IRIS_313-10558, IRIS_313-10559, IRIS_313-10562, IRIS_313-10564, IRIS_313-10567, IRIS_313-10568, IRIS_313-10569, IRIS_313-10570, IRIS_313-10577, IRIS_313-10578, IRIS_313-10580, IRIS_313-10581, IRIS_313-10582, IRIS_313-10583, IRIS_313-10631, IRIS_313-10642, IRIS_313-10644, IRIS_313-10645, IRIS_313-10649, IRIS_313-10653, IRIS_313-10656, IRIS_313-10657, IRIS_313-10660, IRIS_313-10677, IRIS_313-10679, IRIS_313-10689, IRIS_313-10693, IRIS_313-10703, IRIS_313-10704, IRIS_313-10722, IRIS_313-10740, IRIS_313-10741, IRIS_313-10743, IRIS_313-10744, IRIS_313-10745, IRIS_313-10746, IRIS_313-10747, IRIS_313-10758, IRIS_313-10759, IRIS_313-10761, IRIS_313-10765, IRIS_313-10766, IRIS_313-10767, IRIS_313-10770, IRIS_313-10773, IRIS_313-10776, IRIS_313-10780, IRIS_313-10781, IRIS_313-10783, IRIS_313-10784, IRIS_313-10785, IRIS_313-10788, IRIS_313-10789, IRIS_313-10790, IRIS_313-10793, IRIS_313-10794, IRIS_313-10795, IRIS_313-10796, IRIS_313-10798, IRIS_313-10799, IRIS_313-10800
